# Brigade-Surgeon Thomas Edwin Burton Brown

**Published:** 1911-07-22

**Authors:** 


### OBITUARY.

Brigade-Surgeon Thomas Edwin Burton Brown, C.I.E., M.D., formerly Principal of the Lahore Medical College, has died at his home in Brondesbury at the age of 78. After studying at King's College School and at Guy's Hospital, he graduated M.D. of London with honours, being Exhibitioner and Gold Medallist in Anatomy and Materia Medica, and Gold Medallist in Botany, Surgery, and Physiology. For a short time he was Demonstrator of Anatomy at Guy's, but in 1858 he joined the Indian Medical Service, and in 1860 became house surgeon of the Medical College Hospital, Calcutta. He was transferred to the Punjab in 1861, and also lectured in four subjects at the Lahore Medical School. This was transformed into a college in 1870, and he became first Principal and Lecturer on Medicine and Physiology. He officiated as civil surgeon of Lahore on several occasions, and wrote a valuable monograph on the poisons of the Punjab. He retired in 1889 to become medical tutor of the Ayherst Hostel, Cambridge, and in 1891 was created a C.I.E. In 1901 he became Master of the Apothecaries' Society. He was president of the Therapeutical Society in 1906 and a vice-president of the Royal Society of Medicine.

